# Odorant Receptors Mediating Benzaldehyde Detection in *Mythimna separata*: Functional Identification and Ligand Selectivity

**DOI:** 10.3390/insects17101059

**Published:** 2026-10-14

**Authors:** Dongdong Sun, Xiaoqing Wang, Yiping Fan, Bo Zhang, Wenyi Yu, Yang Liu, Guirong Wang

**Affiliations:** 1 Shenzhen Branch, Guangdong Laboratory for Lingnan Modern Agriculture, Synthetic Biology Laboratory of the Ministry of Agriculture and Rural Affairs, Agricultural Genomics Institute at Shenzhen, Chinese Academy of Agricultural Sciences, Shenzhen 518120, China; dongdsyjl@163.com (D.S.); 13045120098@163.com (B.Z.); 2 State Key Laboratory for Biology of Plant Diseases and Insect Pests, Institute of Plant Protection, Chinese Academy of Agricultural Sciences, Beijing 100193, China; 18511602133@163.com (X.W.); fanyp98@126.com (Y.F.); ywy2012@126.com (W.Y.)

**Keywords:** *Mythimna separata*, benzaldehyde, odorant receptor, ligand selectivity, insect olfaction

## Abstract

Benzaldehyde (BA) is a ubiquitous aromatic volatile produced by both plants and insects. In *Mythimna separata*, it also serves as a male-emitted pheromonal signal that mediates male–male competition by antagonizing the pheromone receptor MsepPR3 and disrupting the perception of the female sex pheromone Z11-16:Ald. Whether *M. separata* also possesses conventional odorant receptors (ORs) that detect BA as an environmental volatile remains unknown. Here, we investigated the peripheral molecular basis of BA detection in *M. separata*. Electroantennogram (EAG) recordings revealed robust, dose-dependent responses to BA in both female and male adults, demonstrating that the antennal olfactory system readily detects BA. Candidate ORs were selected based on phylogenetic relationships and functional evidence from homologous receptors in other noctuid moths and subsequently characterized using the *Xenopus* oocyte expression system. Two BA-responsive ORs with distinct ligand profiles were identified. MsepOR42 displayed a highly selective response to BA, whereas MsepOR29 showed broader tuning and responded to BA, benzyl alcohol and 2-phenylethanol. These results establish the peripheral molecular basis of BA detection in *M. separata* and reveal that this ecologically important volatile is encoded by ORs with contrasting ligand selectivity. Our findings expand the understanding of aromatic volatile coding in moths and identify candidate molecular targets for odor-based pest management.

## Simple Summary

Benzaldehyde (BA) is an aromatic volatile shared by plants and insects and can influence diverse insect behaviors. In *Mythimna separata*, BA is also a male-emitted chemical signal that mediates male–male competition by antagonizing the pheromone receptor MsepPR3 and interfering with the perception of the female sex pheromone Z11-16:Ald. Because BA is also a common plant volatile, it remains unclear whether *M. separata* has dedicated odorant receptors (ORs) to detect this ecologically shared compound. Here, we identified two ORs involved in BA perception. MsepOR42 showed a highly selective response to BA, whereas MsepOR29 responded to BA, benzyl alcohol and 2-phenylethanol. These results demonstrate that *M. separata* relies on ORs with different degrees of ligand selectivity to encode an ecologically shared aromatic volatile, providing new insight into the molecular basis of moth olfaction and potential targets for odor-based pest management.

## 1. Introduction

Insects rely on highly sensitive, selective olfactory systems to detect diverse volatile chemical cues in their surroundings, including plant volatiles, sex pheromones, and other semiochemicals. These cues provide critical information for host-plant finding, feeding, oviposition, predator avoidance, and mate localization [1,2,3,4,5]. Volatile odorants are primarily detected by odorant receptors (ORs) expressed on the dendritic membranes of olfactory sensory neurons (OSNs) within antennal sensilla [6,7]. In most insects, ORs form heteromeric complexes with the conserved odorant receptor co-receptor (Orco), generating ligand-gated ion channels that convert odor binding into neuronal signals [8,9,10]. Although pheromone receptors (PRs), a specialized lineage of insect ORs, have received considerable attention in moths, conventional ORs are increasingly recognized as key components of the sensory system that detects ecologically important plant volatiles. Functional analyses using heterologous expression systems, electrophysiological recording, and genetic approaches have consequently provided substantial insight into peripheral odor coding and insect–plant chemical interactions [11,12,13].

Plant-derived volatile organic compounds (VOCs) are a major source of chemical information for herbivorous insects. Released from flowers, leaves, fruits, and other tissues, these compounds can signal plant identity, tissue status, resource quality, and herbivore attack, thereby shaping host-plant localization, feeding, oviposition, and resource-selection behavior [14,15,16,17]. Their effects on insect behavior depend strongly on chemical composition, concentration, and ecological context, with individual compounds or blends acting as attractants, repellents, or context-dependent cues [16,18]. Effective discrimination of these environmental signals is therefore essential for insects navigating chemically complex habitats. In Lepidoptera, conventional ORs form a major molecular basis for detecting such environmental volatiles. Functional characterization of moth ORs has revealed considerable diversity in ligand tuning. Some receptors are highly selective and respond predominantly to a single volatile or a small group of structurally related compounds, whereas others recognize a broader range of chemicals. For example, *Helicoverpa armigera* HarmOR42 responds highly selectively to the aromatic compound phenylacetaldehyde (PAA), whereas HarmOR52 and HarmOR59 recognize multiple host-plant volatiles from different chemical classes [19]. Such differences in receptor tuning enable moths to encode chemically diverse environmental information through a combination of narrowly and broadly responsive ORs. Despite the growing knowledge of plant-volatile receptors, the molecular mechanisms underlying the detection of individual aromatic volatiles remain incompletely understood. In particular, how moths encode compounds that are widespread in plants and associated with multiple ecological functions remains largely unresolved.

Benzaldehyde (BA) is a widespread VOC found in numerous plant species, occurring in flowers, fruits, leaves, and other tissues [20,21,22,23]. In *Petunia hybrida*, BA is produced through a peroxisomal β-oxidation pathway, confirming its endogenous biosynthesis in plants [24]. As a component of plant volatile blends, BA can convey information relevant to plant–insect interactions and influence insect behavior and resource use. Its effects depend on factors including chemical background, concentration, and ecological context. For example, BA contributes to *Callosobruchus chinensis* attraction to host-plant volatile blends [25], while differences in BA abundance among plant cultivars are associated with feeding preferences in *Frankliniella intonsa* [26]. In moths, BA can likewise induce species-specific responses. Plant-derived BA elicits antennal electrophysiological responses in female *Phthorimaea operculella* and has been reported to exert repellent and oviposition-suppressing effects [27]. Conversely, BA can serve as an attractive plant-derived cue for *Grapholita molesta*, influencing male attraction and female oviposition behavior [28,29]. Thus, BA is an ecologically relevant volatile that can contribute to host recognition, resource selection, and oviposition, although its behavioral effects differ among insect species and ecological settings. Despite its widespread distribution and diverse ecological roles, the receptor mechanisms underlying BA detection remain poorly resolved. In *H. armigera*, functional analyses have identified ORs with contrasting responses to BA: HarmOR59 responds to BA within a relatively broad ligand spectrum, whereas HarmOR10 exhibits comparatively narrow tuning and high selectivity for BA [19]. These observations support a role for conventional ORs in BA perception by lepidopteran insects. However, BA-responsive ORs have been functionally characterized in only a small number of moth species, leaving unresolved whether BA recognition relies on conserved receptor mechanisms or species-specific adaptations and how individual receptors differ in ligand selectivity and sensitivity. In particular, the molecular basis of BA detection in the agriculturally important pest *Mythimna* (formerly *Pseudaletia*) *separata* remains unknown.

*M. separata* is a major migratory pest of cereal crops in Asia and causes substantial economic losses during periodic outbreaks [30]. Our previous studies demonstrated that BA is a major component of male-emitted volatiles in *M. separata* and functions as an olfactory antagonist during male–male competition. Specifically, BA competitively inhibits activation of the pheromone receptor MsepPR3 by the female sex pheromone component Z11-16:Ald, thereby disrupting male *M. separata* orientation toward females during courtship competition [31]. These findings established an important role for BA in the chemical communication of *M. separata*, while also raising the question of how this species detects and encodes BA at the peripheral olfactory level. Because BA can evoke diverse ecological responses in insects and moth ORs vary substantially in ligand selectivity and response breadth, identifying the receptors responsible for BA detection in *M. separata* is essential for resolving the molecular basis of its perception. In this study, we systematically identified and functionally characterized BA-responsive ORs by integrating phylogenetic analysis, tissue-specific expression profiling, and heterologous expression in *Xenopus* oocytes coupled with two-electrode voltage-clamp electrophysiology. We further assessed the ligand selectivity and response sensitivity of the identified receptors. Our results provide new insight into the peripheral coding of aromatic plant volatiles and male-emitted chemical signals in noctuid moths and advance the molecular understanding of BA perception in *M. separata*.

## 2. Materials and Methods

### 2.1. Insect Rearing

The *M. separata* population used in this study was originally collected in Xinxiang, Henan Province, China, in September 2020 and has been maintained continuously at the Institute of Plant Protection, Chinese Academy of Agricultural Sciences. Larvae and adults were reared in climate-controlled chambers under separate humidity conditions. Larvae were maintained at 27 ± 1 °C and 40 ± 5% relative humidity (RH), whereas adults were maintained at the same temperature and 65 ± 5% RH; both stages were exposed to a 14 h light:10 h dark photoperiod. Larvae were fed an artificial diet and reared in groups in plastic containers. At the prepupal stage, mature larvae were transferred to plastic boxes containing sandy soil for pupation. Newly emerged adults were supplied with a 10% honey solution, which was replaced daily to support normal development and reproduction.

### 2.2. Chemical Compounds

For electroantennogram (EAG) recordings, benzaldehyde (BA) and the four female sex pheromone components were individually dissolved in n-hexane (Sigma-Aldrich, St. Louis, MO, USA) to prepare 100 μg/μL stock solutions. The four pheromone components were diluted to 10 μg/μL for subsequent assays, whereas BA was further diluted to 10 μg/μL, 1 μg/μL, 100 ng/μL, and 10 ng/μL for dose–response experiments. All solutions were stored at −20 °C until use. For two-electrode voltage-clamp recordings, BA and 28 additional plant volatile compounds were dissolved in dimethyl sulfoxide (DMSO; Sigma-Aldrich, St. Louis, MO, USA) at 1 M and stored at −20 °C. Before electrophysiological assays, the stock solutions were diluted with 1× Ringer’s solution (96 mM NaCl, 2 mM KCl, 5 mM MgCl_2_, 0.8 mM CaCl_2_, and 5 mM HEPES, pH 7.6) to a final concentration of 10^−4^ M. Ringer’s solution containing 0.1% DMSO was used as the negative control. Detailed information on all chemical compounds, including their chemical names and sources, is provided in Table S1.

### 2.3. EAG Recordings

For EAG assays, 10 μL of each test solution was applied to a strip of filter paper (0.5 × 5 cm). After the solvent fully evaporated, the filter paper was inserted into a Pasteur pipette, sealed with Parafilm, and labeled with the compound identity and concentration. Antennae were excised from 4-day-old virgin male or female *M. separata* adults under a stereomicroscope (SZ61; Olympus Corporation, Tokyo, Japan). One antenna was removed at the base, and a small portion of the distal tip was excised to facilitate electrical contact. The antenna was mounted between two glass capillary electrodes filled with conductive saline and connected to silver wire electrodes of an EAG recording system. A continuous stream of humidified, charcoal-filtered air was passed over the antenna throughout the recording. The Pasteur pipette containing the odor stimulus was inserted into a side port of the stimulus delivery tube. After the baseline signal stabilized, the stimulus was delivered by pressing a foot pedal, generating a 0.2 s odor pulse. A minimum 30 s interval was maintained between successive stimuli, and the next stimulus was applied only after the baseline had returned to a stable level.

At the beginning of each recording, n-hexane was presented as the solvent control, followed by sequential presentation of the test compounds. For comparisons among compounds at the same concentration, stimulus order was randomized. For concentration-response assays, stimuli were presented from the lowest to the highest concentration. The identity and concentration of each stimulus were recorded immediately after measurement. Only one antenna from each adult was used. EAG amplitudes were corrected by subtracting the response to the solvent control from the raw response to each test compound: Corrected EAG response = Raw response to the test compound—Response to the solvent control. EAG recording was performed as described previously [4,32].

### 2.4. Sequence Analysis and Phylogenetic Analysis

To determine the phylogenetic relationships of *M. separata* ORs, 63 *M. separata* OR protein sequences were analyzed together with 67 ORs from *H. armigera* and 71 ORs from *Spodoptera frugiperda* [19,33,34,35,36,37]. The OR protein sequences from each species were aligned and trimmed using MAFFT (v7.487) under default parameters [38]. The phylogenetic tree was then constructed with IQ-TREE (v2.1.4-beta) using the options “-m MFP -bb 1000” [39]. The highly conserved Orco subfamily was used as the outgroup for rooting the tree. The resulting phylogeny was visualized and annotated with FigTree v1.4.3. TOPCONS (https://topcons.net/) was used for transmembrane domain (TMD) prediction.

### 2.5. RNA Extraction, cDNA Synthesis and MsepOR Cloning

Antennae, proboscises, labial palps, heads (without antennae, proboscises, or labial palps), thoraces, abdomens, legs, and genitalia were collected from 4-day-old virgin male and female *M. separata* adults. Dissected tissues were immediately frozen in liquid nitrogen and stored at −80 °C until RNA extraction. Total RNA was isolated from each tissue using TRIzol reagent (Invitrogen, Carlsbad, CA, USA) according to the manufacturer’s instructions. RNA concentration was measured with a NanoDrop 2000 spectrophotometer (NanoDrop Technologies, Wilmington, DE, USA), and RNA integrity was assessed by electrophoresis on a 1% agarose gel. First-strand cDNA was synthesized from total RNA using the TransScript One-Step gDNA Removal and cDNA Synthesis SuperMix (TransGen Biotech, Beijing, China) following the manufacturer’s protocol. The resulting cDNA was stored at −20 °C and used for subsequent gene cloning and quantitative real-time PCR (qPCR).

Gene-specific primers were designed from the coding sequences of candidate OR genes identified in *M. separata* (Table S2). Full-length open reading frames (ORFs) of *MsepOR29* (GenBank accession no. MN171142.1) and *MsepOR42* (MN171125.1) were amplified from antennal cDNA. PCR was performed with PrimeSTAR HS DNA Polymerase (Takara, Dalian, China) in a 25 μL reaction containing 1 μL antennal cDNA, 1 μL each of forward and reverse primers, 12.5 μL of 2× PrimeSTAR HS Mix, and nuclease-free water. The amplification program included an initial denaturation at 98 °C for 3 min, followed by 35 cycles of 98 °C for 10 s, 57 °C for 10 s, and 72 °C for 90 s, and a final extension at 72 °C for 2 min. PCR products were purified using an EasyPure Quick Gel Extraction Kit (TransGen Biotech, Beijing, China), ligated into the pEASY-Blunt cloning vector (TransGen Biotech, Beijing, China), and sequenced.

### 2.6. Quantitative Real-Time PCR

The expression profiles of MsepOR29 and MsepOR42 were examined in antennae, proboscises, labial palps, heads (without antennae, proboscises, or labial palps), thoraces, abdomens, legs, and genitalia of 4-day-old virgin male and female *M. separata* adults. *MsepRPS3* was used as the reference gene for normalization [40]. Gene-specific primers were designed from the coding sequences of the two candidate ORs using Primer3Plus (https://www.primer3plus.com/), and the primer sequences are provided in Table S2. Primer amplification efficiencies were determined from standard curves generated using a 5-fold serial dilution of female antennal cDNA. Three independent biological replicates were analyzed for each tissue, with three technical replicates per biological replicate.

Quantitative real-time PCR (qPCR) was performed on an ABI Prism 7500 Fast Real-Time PCR System (Thermo Fisher Scientific, Waltham, MA, USA). Each 20 μL reaction contained 10 μL PerfectStart Green qPCR SuperMix (+DyeII) (TransGen Biotech, Beijing, China), 0.5 μL each of forward and reverse primers (10 μM), 1 μL cDNA template, and 8 μL RNase-free water. The amplification program comprised an initial denaturation at 95 °C for 2 min, followed by 40 cycles of 95 °C for 10 s and 62 °C for 34 s. Relative transcript abundance was calculated using the 2^−ΔΔCt^ method [41], where ΔCt = (Ct, MsepORx − Ct, MsepRPS3), and ΔΔCt = (ΔCt, each sample − mean ΔCt, minimum). The minimum ΔCt within each biological replicate was used as the calibrator, and the mean value of the calibrators from three biological replicates was used as the minimum ΔCt. Differences in antennal expression between males and females were evaluated using the Mann–Whitney U test, with *p* < 0.05 considered statistically significant.

### 2.7. MsepOR Expression in Xenopus Oocytes and Electrophysiological Recordings

The full-length ORFs of *MsepOR29* and *MsepOR42* were individually cloned into the pT7Ts expression vector using the primers listed in Table S2. The MsepOrco construct was derived from the previously reported *M. separata* Orco expression construct [42]. Recombinant plasmids were linearized with the appropriate restriction enzymes, and complementary RNAs (cRNAs) were synthesized using a mMESSAGE mMACHINE^®^ T7 Kit (Ambion, Austin, TX, USA) according to the manufacturer’s instructions. cRNA concentrations were determined with a NanoDrop 2000 spectrophotometer (NanoDrop Technologies, Wilmington, DE, USA) and adjusted to 2 μg/μL. Equal volumes of receptor and MsepOrco cRNAs were mixed at a 1:1 (v/v) ratio and microinjected into healthy, mature *Xenopus* oocytes using a Nanoject II microinjector (Drummond Scientific, Broomall, PA, USA). Each oocyte received 27.6 nL of the cRNA mixture and was subsequently incubated in culture medium at 18 °C for 2–3 days before electrophysiological analysis. Responses of the heterologously expressed receptors to odorant compounds were recorded using two-electrode voltage-clamp electrophysiology with an OC-725C oocyte clamp (Warner Instruments, Hamden, CT, USA). During recording, oocytes were continuously superfused with 1× Ringer’s solution (96 mM NaCl, 2 mM KCl, 5 mM MgCl_2_, 0.8 mM CaCl_2_, and 5 mM HEPES, pH 7.6) and clamped at −80 mV. Test compounds were applied at a final concentration of 10^−4^ M for 15 s, followed by continuous washing with Ringer’s solution until the current returned to the prestimulus baseline before the next application. Electrophysiological signals were acquired using a Digidata 1440A data acquisition system and analyzed with pCLAMP 10.2 software (Molecular Devices, Sunnyvale, CA, USA).

## 3. Results

### 3.1. BA Elicits Antennal EAG Responses

**Figure 1:**
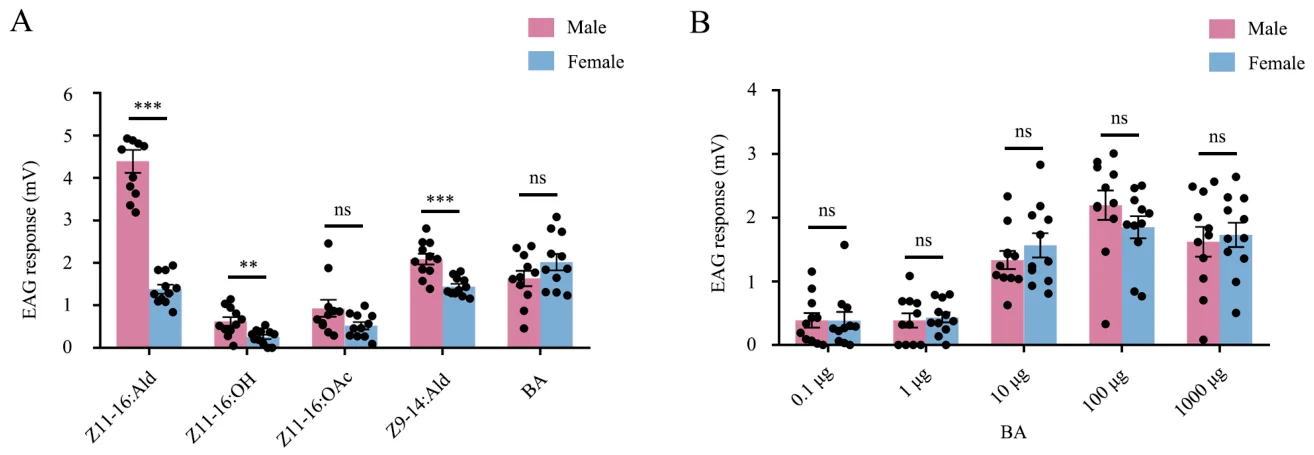
Antennal EAG responses of male and female *M. separata* to BA. (**A**) EAG responses to BA and four sex pheromone components at 100 μg. (**B**) Dose-dependent EAG responses of males and females to BA. Error bars indicate SEM (*n* = 11). Black dots represent individual data points. Differences between sexes were evaluated using the Mann–Whitney U test (ns, not significant; ** *p* < 0.01; *** *p* < 0.001).

We first compared the antennal EAG responses of female and male *M. separata* adults to BA and four female sex pheromone components. At 100 μg, BA elicited strong EAG responses in both sexes, with no significant difference between females and males (Figure 1A). We then examined responses across a concentration series of BA. EAG amplitudes increased progressively with increasing BA concentration, demonstrating a clear dose-dependent response (Figure 1B). No significant differences between the sexes were detected at any concentration tested (Figure 1B). Thus, both female and male *M. separata* adults readily detect BA through their antennae and exhibit comparable peripheral olfactory responses to this compound.

### 3.2. Screening of Candidate ORs for BA

**Figure 2:**
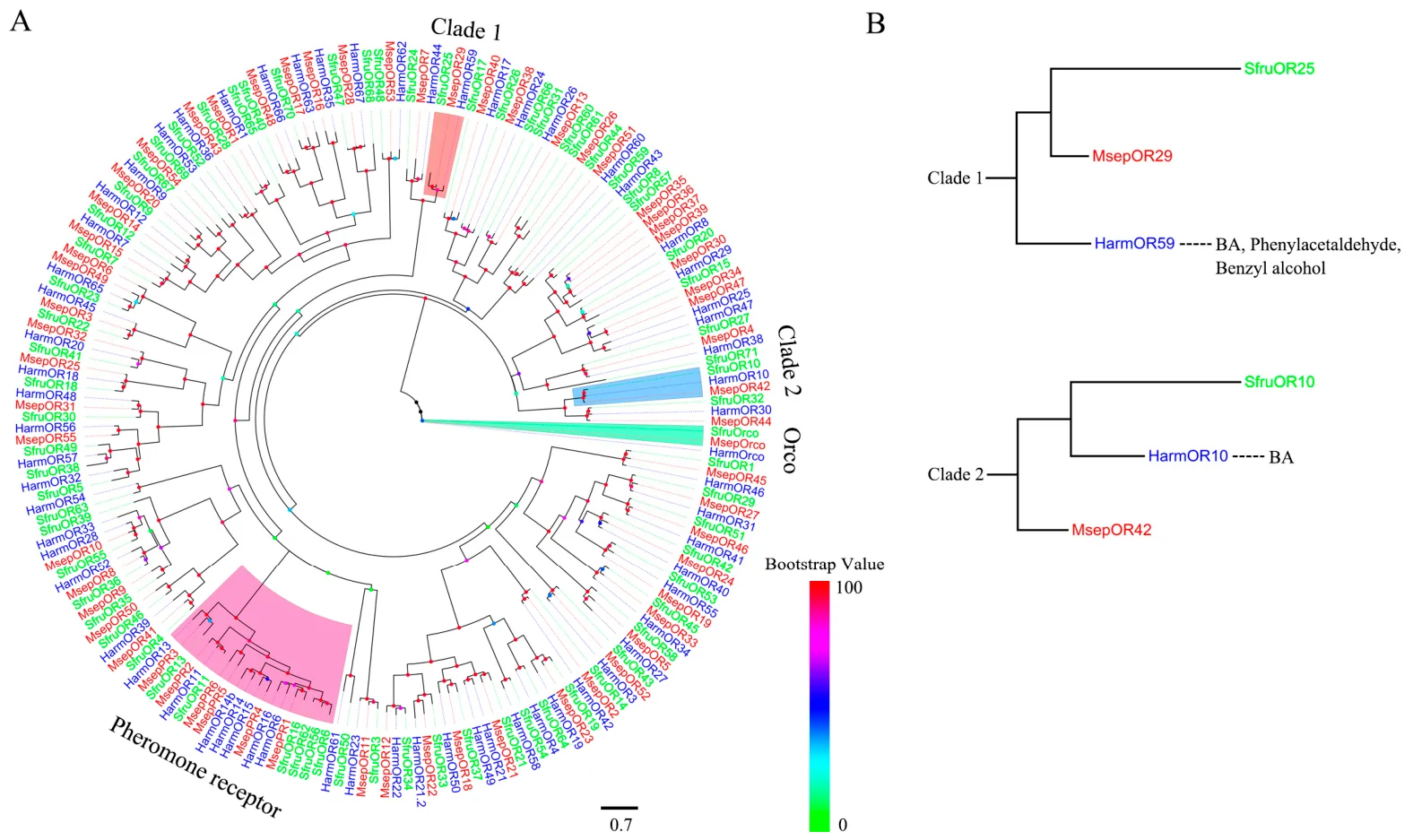
Phylogenetic relationships of ORs from three noctuid species. (**A**) Phylogenetic analysis of ORs from *M. separata*, *Helicoverpa armigera*, and *Spodoptera frugiperda*. (**B**) Two candidate clades containing ORs associated with BA detection. Msep, *M. separata*; Harm, *H. armigera*; Sfru, *S. frugiperda*.

The strong antennal responses to BA in both sexes suggest that its detection may involve one or more antennal-expressed ORs. To identify candidate receptors, we constructed a phylogenetic tree using OR protein sequences from *M. separata*, *H. armigera*, and *S. frugiperda*, together with previously characterized ORs [19]. Orco and pheromone receptors (PRs) formed distinct, well-supported clades, consistent with their established evolutionary relationships (Figure 2A). Within the conventional ORs, two *M. separata* receptors clustered with functionally characterized receptors known to respond to BA or structurally related aromatic volatiles, forming two candidate clades (Clades 1 and 2) (Figure 2A,B).

### 3.3. Gene Cloning

**Figure 3:**
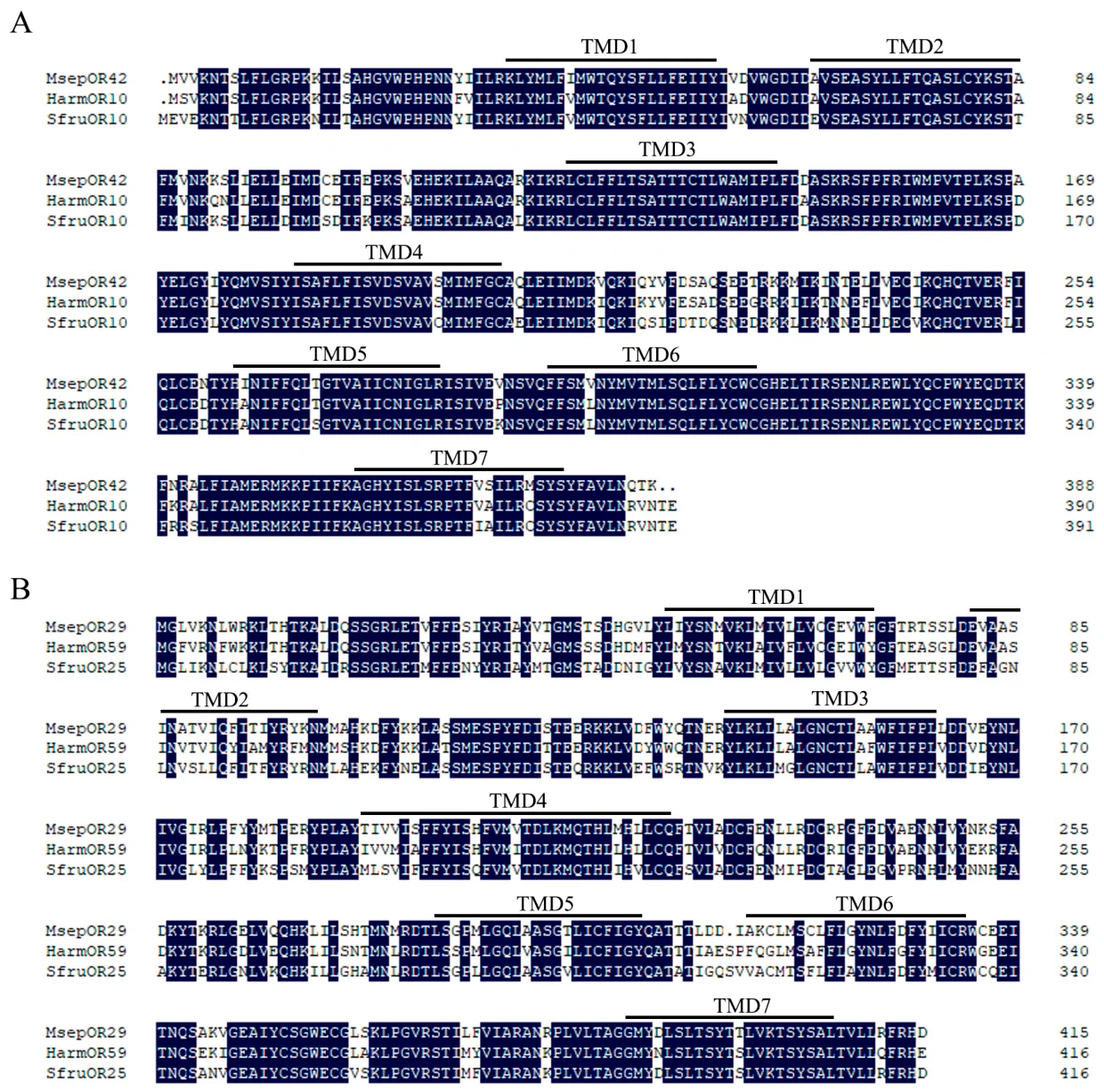
Amino acid sequence alignment of MsepOR29 and MsepOR42 from *M. separata* with their orthologs from other Noctuidae species. (**A**) Alignment of MsepOR42 with HarmOR10 from *H. armigera* and SfruOR10 from *S. frugiperda*. (**B**) Alignment of MsepOR29 with HarmOR59 from *H. armigera* and SfruOR25 from *S. frugiperda*. The seven transmembrane domains (TMD1–TMD7) are indicated by black lines above the alignment. Same amino acid residues in the orthologs are highlighted in black shade.

The full-length coding sequences (CDSs) of *MsepOR29* and *MsepOR42* were successfully amplified and cloned from *M. separata*. *MsepOR29* contained a 1248 bp CDS encoding 415 amino acids, whereas *MsepOR42* comprised 1167 bp and encoded a 388-amino-acid protein. Both receptors were predicted to contain seven transmembrane domains, a characteristic structural feature of insect ORs (Figure 3). Alignment of the corresponding OR29 and OR42 homologs from *M. separata*, *H. armigera*, and *S. frugiperda* revealed high amino acid sequence conservation among the three noctuid species. The sequence identities of OR29 and OR42 homologs were 93.69% and 83.89%, respectively (Figure 3).

### 3.4. Tissue Expression Profiles of MsepOR29 and MsepOR42

**Figure 4:**
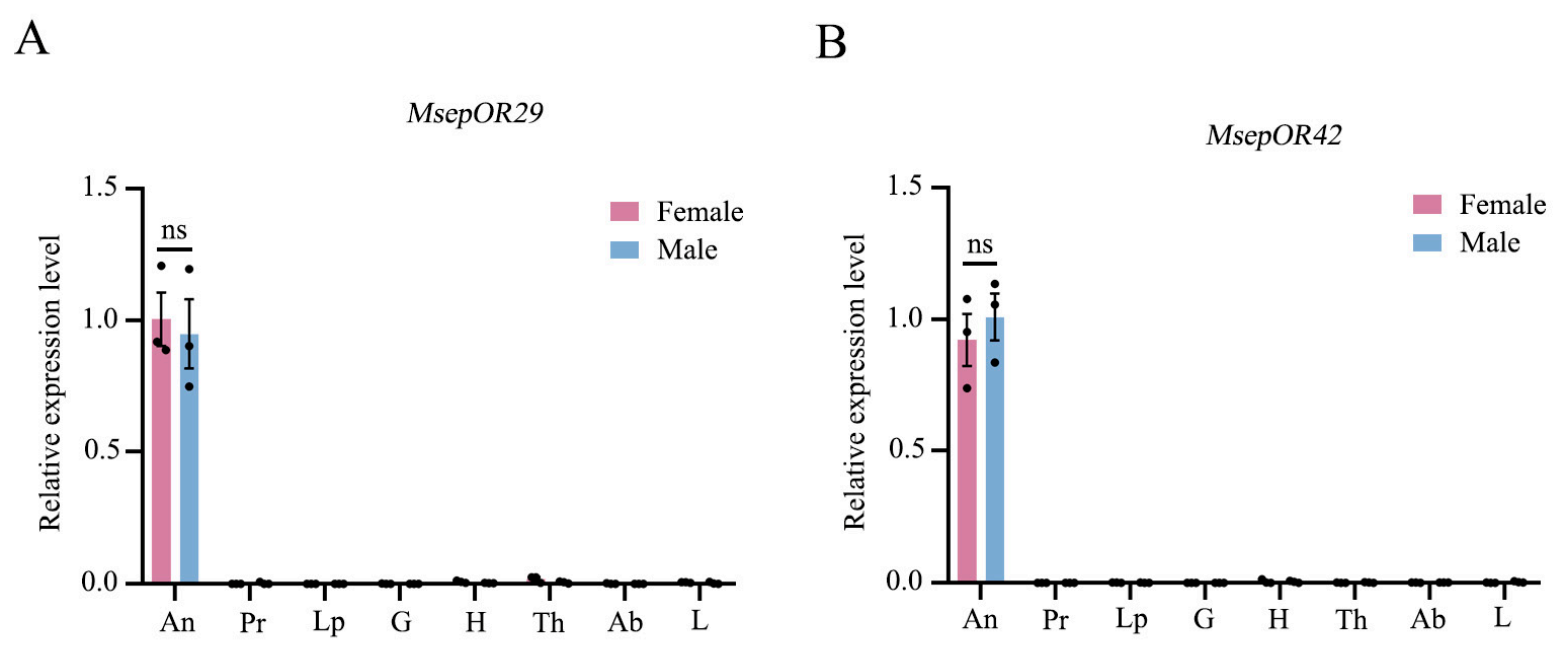
Expression profiles of *MsepOR29* and *MsepOR42* in different tissues. (**A**) Relative expression levels of MsepOR29. (**B**) Relative expression levels of MsepOR42. Error bars indicate SEM (*n* = 3). Black dots represent individual data points. Data were analyzed using the Mann–Whitney U test (ns, no significant difference between males and females). An, antennae; Pr, proboscises; Lp, labial palps; G, genitalia; H, heads; Th, thoraxes; Ab, abdomens; L, legs.

We next examined the tissue distribution of *MsepOR29* and *MsepOR42* transcripts in adult male and female *M. separata* using qPCR. Both genes were expressed predominantly in the antennae, whereas transcript abundance was extremely low or nearly undetectable in the other tissues examined, including the proboscis, labial palps, genitalia, head without antennae, thorax, abdomen, and legs (Figure 4A,B). Antennal expression levels also did not differ significantly between males and females for either gene (Figure 4A,B). The similar antennal expression profiles in the two sexes are consistent with their comparable EAG responses to BA and further support the involvement of MsepOR29 and MsepOR42 in antennal BA detection.

### 3.5. Functional Characterization of Candidate MsepORs

To determine whether MsepOR29 and MsepOR42 function as BA-responsive receptors, each receptor was co-expressed with MsepOrco in *Xenopus* oocytes and tested against a panel of 29 odorants, including BA and structurally diverse volatile compounds, using two-electrode voltage-clamp electrophysiology.

**Figure 5:**
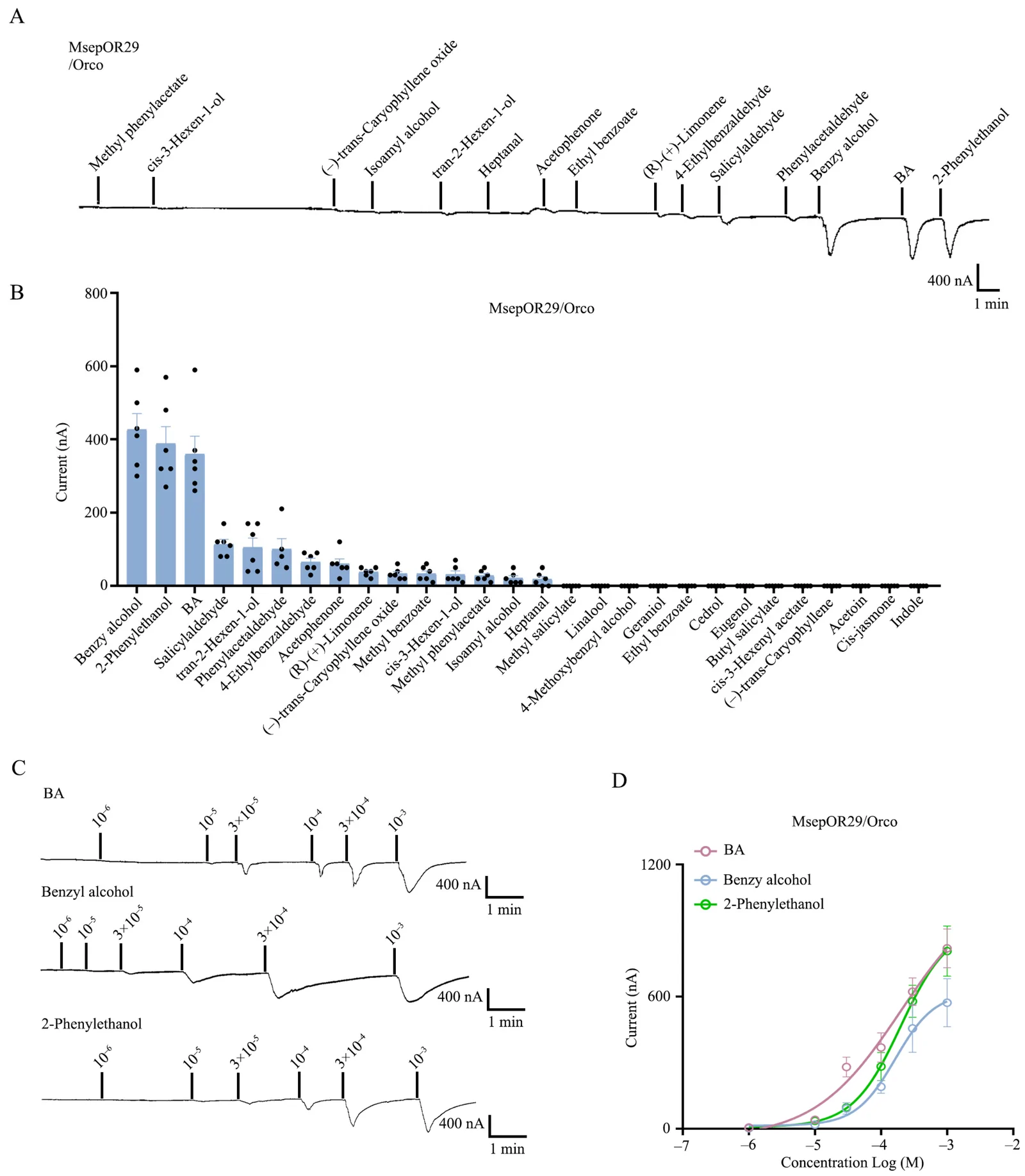
Responses of *Xenopus* oocytes co-expressing MsepOR29 and MsepOrco to BA and related compounds. (**A**) Inward currents elicited by BA and related compounds at 10^−4^ M. (**B**) Response profile of MsepOR29/Orco expressing oocytes. Black dots represent individual data points. (**C**) Inward currents elicited by different concentrations of BA, benzyl alcohol and 2-phenylethanol. (**D**) Dose–response curves of MsepOR29/Orco to BA, benzyl alcohol and 2-phenylethanol. Error bars indicate SEM (*n* = 6).

**Figure 6:**
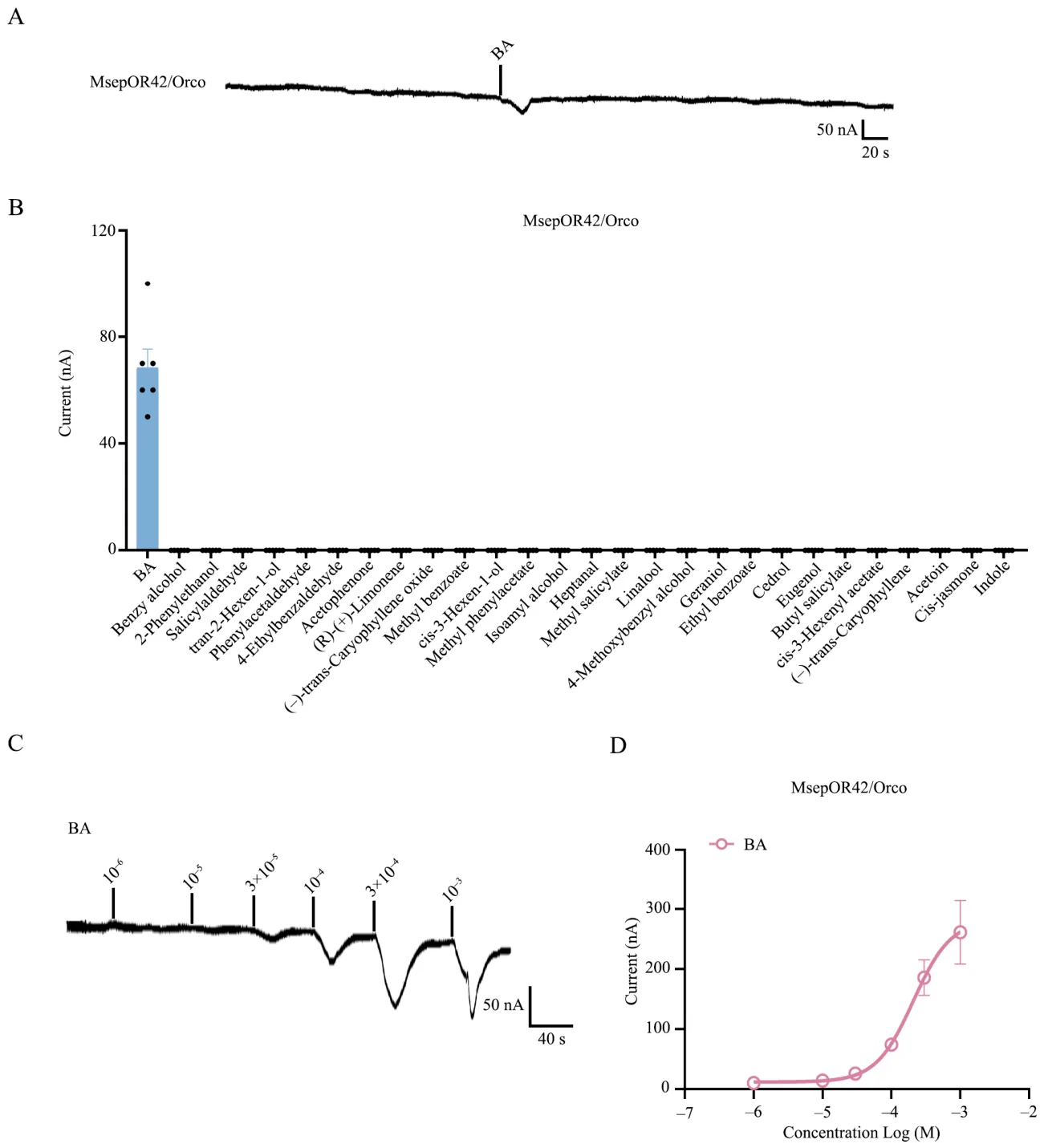
Responses of *Xenopus* oocytes co-expressing MsepOR42 and MsepOrco to BA and related compounds. (**A**) Inward currents elicited by BA and related compounds at 10^−4^ M. (**B**) Response profile of MsepOR42/Orco expressing oocytes. Black dots represent individual data points. (**C**) Inward currents elicited by different concentrations of BA. (**D**) Dose–response curve of MsepOR42/Orco to BA. Error bars indicate SEM (*n* ≥ 6).

At 10^−4^ M, MsepOR29/Orco generated robust inward currents in response to benzyl alcohol, 2-phenylethanol and BA, with mean response amplitudes of 426.7, 388.3, and 360.0 nA, respectively (Figure 5A,B). Thus, MsepOR29 recognizes multiple volatile compounds and exhibits relatively broad ligand tuning. In contrast, MsepOR42/Orco responded only to BA among the compounds tested, producing a mean current of 68.3 nA (Figure 6A,B). These results establish MsepOR29 and MsepOR42 as BA-responsive ORs with markedly different ligand selectivities, suggesting that they may encode BA through distinct contributions to peripheral olfactory coding.

We next determined the concentration dependence of the responses to each receptor’s active ligands. MsepOR29/Orco responded to benzyl alcohol, 2-phenylethanol, and BA in a concentration-dependent manner, with estimated EC_50_ values of 1.641 × 10^−4^, 1.991 × 10^−4^, and 2.024 × 10^−4^ M, respectively (Figure 5C,D). Similarly, MsepOR42/Orco displayed a concentration-dependent response to BA, with an EC_50_ value of 2.086 × 10^−4^ M (Figure 6C,D). Together, these results identify MsepOR29 and MsepOR42 as two BA-responsive odorant receptors in *M. separata*, with MsepOR29 displaying broader ligand tuning and MsepOR42 exhibiting pronounced selectivity for BA.

## 4. Discussion

In this study, we identified and functionally characterized two ORs involved in BA detection in *M. separata*. The distinct ligand-tuning profiles of MsepOR29 and MsepOR42 suggest that BA detection may involve multiple ORs with complementary ligand specificities rather than a single dedicated receptor. This diversity may facilitate the detection and discrimination of BA and structurally related aromatic volatiles in complex odor environments, providing a molecular basis for BA perception in noctuid moths.

The ecological significance of BA detection may be related to its occurrence in both plant- and insect-derived chemical environments, where it can convey different types of ecological information [43,44,45,46,47,48]. In several moth species, BA is a constituent of floral and host-plant volatile blends and may contribute to odor-guided foraging. For example, BA is a component of floral odor blends that elicit flight and feeding responses in *Manduca sexta* [49]. In addition, BA alone has been shown to attract male *G. molesta*, and its combination with the species-specific sex pheromone enhances male attraction in a season-dependent manner [28]. These findings suggest that BA can function as an ecologically relevant plant-associated cue, raising the possibility that MsepOR29 and MsepOR42 may contribute to detecting BA-containing floral or host-plant odor blends in *M. separata*. Conversely, BA can also originate from conspecific insects and participate in reproductive communication. In our previous study, male *M. separata* released BA during courtship competition, and BA interfered with rival males’ detection of the female sex pheromone component Z11-16:Ald through antagonistic effects on MsepPR3 [31]. Earlier work further identified BA as the major component of the male pheromone-releasing organ in *M. separata* and suggested that it may act as an arrestant by reducing female escape responses during close-range courtship [50]. Together, these observations suggest that BA may serve dual ecological roles as both a plant-associated odor cue and a potential reproductive signal in *M. separata*.

The distinct response profiles of MsepOR42 and MsepOR29 further prompted comparison with previously characterized BA-responsive ORs from other noctuid species. In *H. armigera*, HarmOR10 responds narrowly and selectively to BA, whereas HarmOR59 has a broader tuning profile and responds strongly to BA together with several aromatic and aliphatic volatiles [19]. A comparable division of receptor tuning was observed in *M. separata*: MsepOR42 was highly selective for BA, whereas MsepOR29 also recognized other structurally related aromatic compounds. The similar BA-selective profiles of MsepOR42 and HarmOR10 suggest that BA recognition may be functionally conserved among noctuid moths, potentially reflecting selection for maintaining sensitivity to an ecologically relevant volatile encountered repeatedly in their chemical environment. By contrast, the broader response profiles of HarmOR59 and MsepOR29 differed substantially in both response magnitude and ligand breadth. HarmOR59 responded strongly to BA, phenylacetaldehyde and several structurally unrelated compounds, including (E)-2-hexen-1-ol, whereas MsepOR29 produced comparatively weaker responses to its additional ligands and showed a more restricted tuning spectrum. These differences indicate that homologous ORs can retain recognition of a common ecologically relevant ligand while undergoing species-specific changes in ligand affinity, sensitivity, and tuning breadth. Previous studies have shown that even relatively small amino acid substitutions, particularly in ligand-interacting regions, can substantially modify these functional properties [51,52,53,54]. The comparison of MsepOR42/MsepOR29 with HarmOR10/HarmOR59 therefore supports a model in which OR evolution preserves recognition of ecologically important odorants while allowing lineage-specific refinement of receptor tuning.

The detection of the same odorant by multiple receptors does not necessarily imply functional redundancy. Instead, overlapping receptor sensitivities may provide complementary channels for odor representation in the peripheral olfactory system [55,56]. Although many moth pheromone receptors (PRs) exhibit strong ligand specificity [57,58], a strict one-to-one correspondence between pheromone components and receptors is not universal [59,60]. In *S. frugiperda*, for example, each of the pheromone components Z7-12:OAc and Z9,E12-14:OAc is recognized by two distinct PRs, demonstrating that a single pheromone component can be encoded through multiple receptor pathways [61,62]. Similar ligand overlap occurs among conventional ORs. For instance, both CvirOR27 and CvirOR43 in *Chloridea virescens* respond to methyl salicylate (MeSA) [63], illustrating that receptor specialization and overlapping ligand recognition can coexist within the same olfactory system. In *M. separata*, the detection of BA by both MsepOR42 and MsepOR29 may likewise reflect complementary rather than redundant receptor functions. Their distinct ligand sensitivities could enable the olfactory system to detect BA across a broader chemical background and contribute to quantitative or contextual encoding of this volatile.

Despite these findings, the present study cannot determine whether MsepOR29 and MsepOR42 preferentially detect plant-derived BA, male-derived BA, or both, because their functions were characterized only in a heterologous expression system. Future studies combining receptor-specific RNAi or CRISPR/Cas9-mediated knockout with antennal electrophysiological recordings, such as EAG and single-sensillum recording (SSR), will be important for establishing the in vivo contribution of MsepOR29 and MsepOR42 to BA detection. Behavioral assays using plant or floral odors, synthetic BA, and male-derived odor sources could further determine whether disruption of either receptor affects host-plant foraging, close-range courtship, or mating acceptance. Such studies would help distinguish the ecological functions of these two receptors and clarify how their complementary ligand tuning contributes to the representation of BA across different behavioral contexts.

## Data Availability

All the data and resources generated for this study are included in the article and the Supplementary Materials.

## Supplementary Materials

The following supporting information can be downloaded at https://www.mdpi.com/article/10.3390/insects17101059/s1, Table S1: The list of chemicals used in this study. Table S2: Primers used in this study.
