# Supplementary material for: Odorant Receptors Mediating Benzaldehyde Detection in Mythimna separata: Functional Identification and Ligand Selectivity
Source: Insects. 2026 Oct 14;17(10):1059. doi: 10.3390/insects17101059 (PMC13705561; doi:10.3390/insects17101059)
Supplement: Supplementary file 1 [file insects-17-01059-s001.zip › insects-4561913-supplementary.pdf]

Table S1. The list of chemicals used in this study.

**Chemicals**	Sources	CAS Numbers
(Z)-11-hexadecenal (Z11-16:Ald)	Nimrod Inc, Changzhou, China	53939-28-9
(Z)-9-tetradecenal (Z9-14:Ald)	Nimrod Inc, Changzhou, China	53939-27-8
(Z)-11-hexadecen-1-ol (Z11-16:OH)	Nimrod Inc, Changzhou, China	56683-54-6
(Z)-11-hexadecen-1-yl acetate (Z11-16:OAc)	Nimrod Inc, Changzhou, China	34010-21-4
Indole	Sigma-Aldrich, St. Louis, MO, USA	120-72-9
Benzaldehyde	Sigma-Aldrich, St. Louis, MO, USA	100-52-7
Benzyl alcohol	Sigma-Aldrich, St. Louis, MO, USA	100-51-6
Phenylacetaldehyde	Sigma-Aldrich, St. Louis, MO, USA	122-78-1
Acetophenone	Sigma-Aldrich, St. Louis, MO, USA	98-86-2
Methyl benzoate	Sigma-Aldrich, St. Louis, MO, USA	93-58-3
Methyl salicylate	Sigma-Aldrich, St. Louis, MO, USA	119-36-8
Linalool	Sigma-Aldrich, St. Louis, MO, USA	78-70-6
4-Methoxybenzyl alcohol	Sigma-Aldrich, St. Louis, MO, USA	105-13-5
4-Ethylbenzaldehyde	Sigma-Aldrich, St. Louis, MO, USA	4748-78-1
Salicylaldehyde	Sigma-Aldrich, St. Louis, MO, USA	90-02-8
2-Phenylethanol	Sigma-Aldrich, St. Louis, MO, USA	60-12-8
(R)-(+)-Limonene	Sigma-Aldrich, St. Louis, MO, USA	5989-27-5
Geraniol	Sigma-Aldrich, St. Louis, MO, USA	106-24-1
Ethyl benzoate	Sigma-Aldrich, St. Louis, MO, USA	93-89-0
Methyl phenylacetate	Sigma-Aldrich, St. Louis, MO, USA	101-41-7
Cedrol	Sigma-Aldrich, St. Louis, MO, USA	77-53-2
Isoamyl alcohol	Sigma-Aldrich, St. Louis, MO, USA	123-51-3
Eugenol	Sigma-Aldrich, St. Louis, MO, USA	97-53-0
Butyl salicylate	Sigma-Aldrich, St. Louis, MO, USA	2052-14-4
1-Octen-3-ol	Sigma-Aldrich, St. Louis, MO, USA	3391-86-4
Heptanal	Sigma-Aldrich, St. Louis, MO, USA	111-71-7
cis-3-Hexen-1-ol	Sigma-Aldrich, St. Louis, MO, USA	928-96-1
trans-2-Hexen-1-ol	Sigma-Aldrich, St. Louis, MO, USA	928-95-0
cis-3-Hexenyl acetate	Sigma-Aldrich, St. Louis, MO, USA	3681-71-8
(–)-trans-Caryophyllene	Sigma-Aldrich, St. Louis, MO, USA	87-44-5
(–)-trans-Caryophyllene oxide	Sigma-Aldrich, St. Louis, MO, USA	1139-30-6
Acetoin	Sigma-Aldrich, St. Louis, MO, USA	513-86-0
Jasmone	Sigma-Aldrich, St. Louis, MO, USA	488-10-8

Table S2. Primers used in this study.

Primer name	Sequence (5'-3')
Gene cloning	
MsepOR29-C-F	ATGGGTCTCGTCAAAAATCTTTG
MsepOR29-C-R	TTAGTCGTGCCGGAATCGAA
MsepOR42-C-F	ATGGTAGTAAAAAACACATCTTTATTCCT
MsepOR42-C-R	TTATTTCGTCTGATTCAATACTGCAA
qPCR	
MsepRPS3-qPCR-F	AATGAGTTCTTGACCAGGGAG
MsepRPS3-qPCR-R	GTGTCCTCGTCGCCATAAT
MsepOR29-qPCR-F	ACGATGAACATGCGCGACAC
MsepOR29-qPCR-R	TCCTGGCAGTTTGGAGAGGC
MsepOR42-qPCR-F	AGTGGATGTTTGGGGCGACA
MsepOR42-qPCR-R	CAGCGGTATCATGGCCCAGA
Vector construction	
MsepOR29-V-F	atcactagtgggcccGCCACCATGGGTCTCGTCAAAAATCTTTG
MsepOR29-V-R	ctagtcagtcgcggccgcTTAGTCGTGCCGGAATCGAA
MsepOR42-V-F	atcactagtgggcccGCCACCATGGTAGTAAAAAACACATCTTTATTCCT
MsepOR42-V-R	ctagtcagtcgcggccgcTTATTTCGTCTGATTCAATACTGCAA
